# The Hepatitis B Virus Genotype Affects the Persistence of Viral Replication in Immunodeficient NOG Mice

**DOI:** 10.1371/journal.pone.0144775

**Published:** 2015-12-14

**Authors:** Yoshinobu Yokoyama, Takuya Miyagi, Hayato Hikita, Teppei Yoshioka, Kaori Mukai, Takatoshi Nawa, Ryotaro Sakamori, Kazuyoshi Ohkawa, Naoki Hiramatsu, Takeshi Takahashi, Hiroshi Suemizu, Akihide Ryo, Tomohide Tatsumi, Tetsuo Takehara

**Affiliations:** 1 Department of Gastroenterology and Hepatology, Osaka University Graduate School of Medicine, Suita, Osaka, Japan; 2 Laboratory Animal Research Department, Central Institute for Experimental Animals, Kawasaki, Kanagawa, Japan; 3 Department of Microbiology, Yokohama City University School of Medicine, Yokohama, Kanagawa, Japan; Chiba University, Graduate School of Medicine, JAPAN

## Abstract

**Background & Aims:**

At least eight genotypes of Hepatitis B virus (HBV) have been identified. HBV genotype C is the most common genotype in Japan, although the incidence of HBV genotype A is increasing. The reason underlying the differences in viral multiplication of the HBV genotypes is unclear, especially *in vivo*. The purpose of this study was to elucidate the differences in HBV load and the persistence of viremia *in vivo* between genotypes A and C.

**Methods:**

Immunodeficient NOG mice were transfected by hydrodynamic injection with the HBV expression plasmids pHBA1.2 or pHBC1.2, which contain overlength (1.2-mer) copies of the genomes of HBV genotype A or C, respectively.

**Results:**

One day after transfection, the number of HBcAg-positive hepatocytes and serum HBV DNA levels were similar between mice transfected with pHBA1.2 and pHBC1.2. Serum levels of HBV DNA, HBsAg and HBeAg in mice transfected with pHBA1.2 were maintained over 5 months. In contrast, those in mice with pHBC1.2 gradually decreased over time and reached undetectable levels within 3 months after transfection. HBcAg-stained hepatocytes were detected in mice transfected with pHBA1.2, but not pHBC1.2, 5 months post-transfection. Double-staining immunohistochemistry revealed that the number of cleaved caspase3-stained, HBcAg-positive hepatocytes in the pHBC1.2-transfected mice was higher than in the pHBA1.2-transfected mice 3 days post-transfection. Moreover, the plasmid DNA and covalently closed circular DNA levels were decreased in the livers of pHBC1.2-transfected mice. These results suggested that hepatocytes expressing HBV genotype C were eliminated by apoptosis in the absence of immune cells more often than in hepatocytes expressing HBV genotype A.

**Conclusions:**

Immunodeficient mice transfected with HBV genotype A develop persistent viremia, whereas those transfected with HBV genotype C exhibit transient viremia accompanied by apoptosis of HBV-expressing hepatocytes. This differences may affect the clinical courses of patients infected with HBV genotypes A and C.

## Introduction

Hepatitis B virus (HBV) infection is one of the most common viral infections and is a worldwide health problem [1]. At least eight genotypes of HBV have been classified, with the proportion of genotypes varying depending on the region [2]. HBV genotype C was the most common genotype in Japan [3–5], whereas HBV genotype A was rare. However, the proportion of HBV genotype A (especially genotype A2) is increasing in Japan, mainly via sexual transmission [6–9]. HBV genotype A develops into a persistent infection more often than genotype C [9, 10]. Ito K et al. [9] reported that the maximum serum HBV DNA levels were higher among patients with acute HBV genotype A infection compared to those infected with HBV genotype C. However, the mechanisms underlying the differences in the persistence and viral loads of HBV genotypes A and C are unclear. Many studies have shown that immune cells are involved in HBV infection and that immunosuppressive or immunotolerable responses may differ among HBV genotypes [11–14]. Sugiyama M et al. [15] reported that HBV replication speed was slower and the level of hepatitis B surface antigen (HBsAg) in cultured medium was higher for genotype A compared to genotype C *in vitro*; these differences may contribute to differences in HBV DNA level among patients infected with HBV. Ito K et al. [16] proposed that differences in the hepatitis B e antigen (HBeAg) component of genotype A would induce immune tolerance to HBV infection. Based on these reports, the differences in HBV DNA levels between genotypes might be explained by different immune system responses. However, whether immune system-independent mechanisms affect HBV DNA levels *in vivo* is unclear.

Here, we demonstrated that while immunodeficient NOG mice transfected with HBV genotype A exhibited persistent viremia, the HBV DNA levels in mice transfected with HBV genotype C decreased to undetectable levels. In this study, we generated HBV genotype A and C expression plasmids from the same backbone vector. The plasmids were transfected by hydrodynamic injection [17, 18] into NOG mice that lacked T cells, B cells and NK cells to drive expression in murine hepatocytes. Although the number of hepatitis B core antigen (HBcAg)-positive hepatocytes and the serum viral loads were similar 1 day after transfection with the HBV genotype A and C plasmids, the HBcAg-positive hepatocytes and serum HBV DNA levels decreased to undetectable levels after 1 and 3 months, respectively, in NOG mice transfected with HBV genotype C but not genotype A. We showed that the frequency of cleaved caspase-3-positive hepatocytes among HBV-expressing hepatocytes was high in the livers of mice infected with HBV genotype C, suggesting that the expression of HBV genotype C in hepatocytes frequently led to hepatocyte apoptosis in the absence of immune cells. This difference in hepatocyte apoptosis may contribute to the differences in viral load and the persistence of infection. This is the first report that transfection of HBV genotype C by hydrodynamic injection into mice failed to persistent infection even in the absence of immune cells.

## Methods

### Plasmids

We used the plasmid pHBA1.2, which contains an overlength (1.2-mer) copy of HBV genotype A DNA, and plasmid pHBC1.2, which contains an overlength (1.2-mer) copy of HBV genotype C DNA (S1 Fig). The HBV genotype A sequence was cloned from the adw2 subtype (DDBJ/EMBL/GenBank Accession No. X02763), while the HBV genotype C sequence was cloned from the adr4 subtype (DDBJ/EMBL/GenBank Accession No. LC090200) [19]. The insert in pHBA1.2 covered nucleotides 2809-3221/1-3221/1-172 of the adw2 subtype, while the insert in pHBC1.2 covered nucleotides 1400-3215/1-1988 of the adr4 subtype. These plasmids are capable of undergoing transcription and replication of HBV DNA depending on the presence of the HBV promoter sequences in transfected cells. pBluescriptⅡSK+ (pBS) was used as the backbone of the pHBA1.2 and pHBC1.2 plasmids and was used as a control in our *in vivo* study. The reporter plasmid pCMV-SEAP encoding the secreted alkaline phosphatase (SEAP) gene was used as a control in the *in vitro* study. Plasmid DNA was prepared using an EndoFree plasmid system (Qiagen, Venlo, Netherlands) according to the manufacturer’s instructions.

### Cell culture and plasmid transfection

Huh7 cells (a human hepatocellular carcinoma cell line) were obtained from the Japanese Collection of Research Bioresources Cell Bank (Ibaraki, Japan). They were cultured in Dulbecco’s modified Eagle’s medium with 10% fetal bovine serum at 37°C in a 5% CO2 atmosphere. For transfection, 1×10^5^ Huh7 cells were seeded into a 12-well culture dish and transfected with 1 μg of various HBV-expression plasmids with or without 0.1 μg of pCMV-SEAP using the FuGENE HD reagent (Roche Diagnostics, Basel, Switzerland). After transfection, the culture media were changed on days 1, 3 and 6. The transfection efficiency was evaluated by measuring the secreted alkaline phosphatase (ALP) activity using culture media collected on day 1. ALP was measured with the LabAssay^TM^ ALP (Wako, Osaka, Japan) according to the manufacturer’s instructions. The caspase-3/7 activity of the culture medium was measured by Caspase-Glo ^®^ 3/7 assay (Promega Corporation, Madison, WI, USA) according to manufacturer’s instructions. To inhibit apoptosis, we used 10 μM of pan caspase inhibitor, Z-VAD-FMK (Promega Corporation).

### Mice and hydrodynamic injection of the plasmids

We used specific-pathogen-free male NOG (NOD/Shi-*scid*/IL-2Rγ^null^) mice as previously described [20]. The mice were used at the age of 6–8 weeks. To generate an HBV-infected humanized mouse liver as a positive control for cccDNA, we inoculated serum from a chronic hepatitis B patient into a humanized liver Tk-NOG mouse as described previously [21]. All procedures were carried out according to protocols approved by the Institutional Review Board for Clinical Research at Osaka University Hospital. We obtained written informed consent form the patient prior to participation in this study. All mice in this study were housed under conditions of controlled temperature and light with free access to food and water at the Institute of Experimental Animal Science, Osaka University Graduate School of Medicine, and the Central Institute for Experimental Animals (CIEA). All animal procedures were performed according to the Guidelines for Animal Care at the Institutes after approval by the Animal Care and Use Committee of Osaka University Medical School and the Animal Care Committee of CIEA. For plasmid transfection by hydrodynamic injection, 25 μg (3.4×10^18^ copies/body) of plasmid DNA was diluted with 2.0 ml of lactated Ringer’s solution and injected into circulation via the tail vein within 10–20 seconds [17]. Serum was collected from mice and the mice were sacrificed by intraperitoneal injection of pentobarbital (200 mg/kg) at the indicated time points.

### Immunohistochemistry

For the immunohistochemical detection of HBcAg, liver tissues were fixed with 10% neutral buffered formalin and embedded in paraffin. Sections with a thickness of 4 μm were incubated with an anti-HBcAg antibody (Dako, Glostrup, Denmark, B0586) followed by immunoperoxidase staining using the DAB Peroxidase Substrate Kit (Vector Laboratories, Burlingame, CA, USA). For immunofluorescence staining of HBcAg and cleaved caspase-3, the tissues were fixed with 10% neutral buffered formalin and embedded in paraffin. Sections with a thickness of 4 μm were incubated with an in-house mouse monoclonal anti-HBcAg antibody (diluted 1:100) and an anti-cleaved caspase-3 rabbit antibody (diluted 1:300, Cell Signaling Technology, Danvers, MA, USA) as primary antibodies and Alexa Fluor 488 goat anti-mouse IgG (diluted 1:500, Cell Signaling Technology) and Alexa Fluor 555 goat anti-rabbit IgG (diluted 1:500, Cell Signaling Technology) as secondary antibodies. We used VECTASHIELD mounting medium with DAPI (Vector Laboratories) for mounting. The Apoptag^®^ Peroxidase In Situ Apoptosis Detection Kit (Merck Millipore, Billerica, MA, USA) was used for terminal deoxynucleotidyl transferase-mediated deoxyuridine triphosphate nick-end labeling (TUNEL) staining of mouse liver sections according to the manufacturer’s instructions.

### Measuring HBV DNA, HBsAg and HBeAg

A total of 10 μl of supernatant or serum was treated with DNase I (Takara Bio Inc. Shiga, Japan) and then proteinase K. DNA was extracted using a QIAamp DNA Blood Isolation System (Qiagen) according to the manufacturer’s instructions. An HBV DNA-specific sequence was amplified with the Applied Biosystems 7900HT Fast Real-Time PCR System using a sense primer (5’-ACATCAGGATTCCTAGGACCC-3’), antisense primer (5’-GGTGAGTGATTGGAGGTTGG-3’) and FAM probe (5’-CAGAGTCTAGACTCGTGGTGGACTTC-3’) as previously described [22]. To generate standard curves, we used HBV-infected patient serum with a known quantity of HBV DNA; the samples were handled in the same manner as described above. The lower limit of detection was 3.3 log copies/ml. The HBsAg and HBeAg were measured using a chemiluminescent immunoassay (CLIA System, Abbott Laboratories, North Chicago, IL, USA).

### Detection of closed circular DNA (cccDNA) and plasmid DNA in the liver

We extracted a total of 10–40 μg DNA from approximately 30 mg of liver using a QIAamp DNA Mini kit (Qiagen) according to the manufacturer’s instructions. We performed PCR reactions using 200 ng of the extracted DNA. The cccDNA and plasmid DNA were amplified using a sense primer (5’-TTGTGGGTCTTTTGGGCTTT-3’) and antisense primer (5’-ATAGGGGCATTTGGTGGTCT-3’). We performed the PCR amplification in a thermocycler with a cycling program consisting of a denaturation step at 94°Cfor 2 minutes and 35 cycles of 10 seconds at 98°C, 30 seconds at 64°C, and 90 seconds at 72°C. The PCR products were analyzed on a 1% agarose gel by electrophoresis. The detected cccDNA (1320 bp) and plasmid DNA (4910 bp) were quantified using ImageJ software (National Institutes of Health, Bethesda, MD, USA). The intensity of each band was calibrated based on the intensity of the 2000 bp marker. For a negative control, we used the sera of chronic hepatitis B patients according to the procedure approved by the Institutional Review Board for Clinical Research at Osaka University Hospital. We obtained written informed consent form those patients to participate in this study. We extracted DNA from 10 μl of serum collected from an HBV genotype A-infected patient whose serum HBV DNA level was 9.3 log copies/ml and from an HBV genotype C-infected patient whose serum HBV DNA level was 7.4 log copies/ml. One tenth of the total extracted DNA was subjected to PCR as a negative control for the cccDNA. For a positive control for cccDNA, we used extracted DNA from the liver of HBV genotype C-infected humanized liver mouse, whose serum HBV-DNA level was 8.1 log copies/ml. We confirmed the linear standard curve of the band intensity levels used between 12.5 and 200 ng of the extracted DNA (S2A Fig). For the quantification of plasmid DNA, the DNA was amplified using the Applied Biosystems 7900HT Fast Real-Time PCR System with the AmpR probe Mr00661613-cn (Thermo Fisher Scientific Inc., Waltham, MA, USA). Tert (Thermo Fisher Scientific Inc.) was used as an endogenous reference gene.

### Real-time RT-PCR

Mouse liver mRNA was extracted using an RNeasy Mini kit (Qiagen) and converted into complementary DNA with reverse transcriptase (ReverTra Ace^®^ qPCR RT Master Mix; TOYOBO, Osaka, Japan) according to the manufacturer’s instructions. Next, we used Taqman primers to amplify the cDNA using the Applied Biosystems 7900HT Fast Real-Time PCR System (Thermo Fisher Scientific Inc.). The following probes from Thermo Fisher Scientific Inc. were used: β-actin, Mm00607939-s1; IFN-a4, Mm00833969-s1; IFN-b1, Mm00439552-s1; Mx1, Mm01217998-m1; Oas1a, Mm00836412-m1; ISG15, Mm01705338-s1; and IFIT1, Mm00515153-m1. All expression levels were normalized to the β-actin expression level.

### Western blot analysis

Approximately 30 mg of mouse liver was homogenized and centrifuged at 16,500 g for 20 minutes at 4°C. After adding sodium dodecyl sulfate (SDS) sample buffer, the supernatants were boiled for 10 minutes at 95°C. Protein concentrations in the samples were determined by the BCA assay (Thermo Fisher Scientific Inc.). Then, appropriate amounts of protein were loaded onto 8–12% SDS–polyacrylamide gels and electrotransferred onto a polyvinylidene fluoride membrane. The membrane was blocked with 5% skimmed milk or 1% bovine serum albumin for 1 hour at room temperature and then incubated overnight at 4°C with the primary antibodies anti-STAT1, anti-phospho-STAT1, anti-IRF3, anti-phospho-IRF3 (diluted 1:1000, Cell Signaling Technology), anti-STAT2, anti-phospho-STAT2 (diluted 1:1000, Abcam, Cambridge, UK) or β-actin (diluted 1:10,000, Sigma-Aldrich, St. Louis, MO, USA). After the membrane was washed three times for 5 minutes in TBST, it was incubated with the appropriate HRP-conjugated secondary antibody (diluted 1:5000 in TBST) for 1 hour at room temperature. Blotted protein bands were visualized by enhanced chemiluminescence (Thermo Fisher Scientific Inc.) and exposed to X-ray film.

### Statistical analysis

All data were presented as the means ± SEM. The measurements were subjected to Student’s t-test or one-way ANOVA followed by Tukey’s post hoc test. Otherwise, we specified the statistical method that was used in the Fig legend. P < 0.05 was considered statistically significant.

## Results

### Generation of HBV genotype A and C expression plasmids

**Fig 1:**
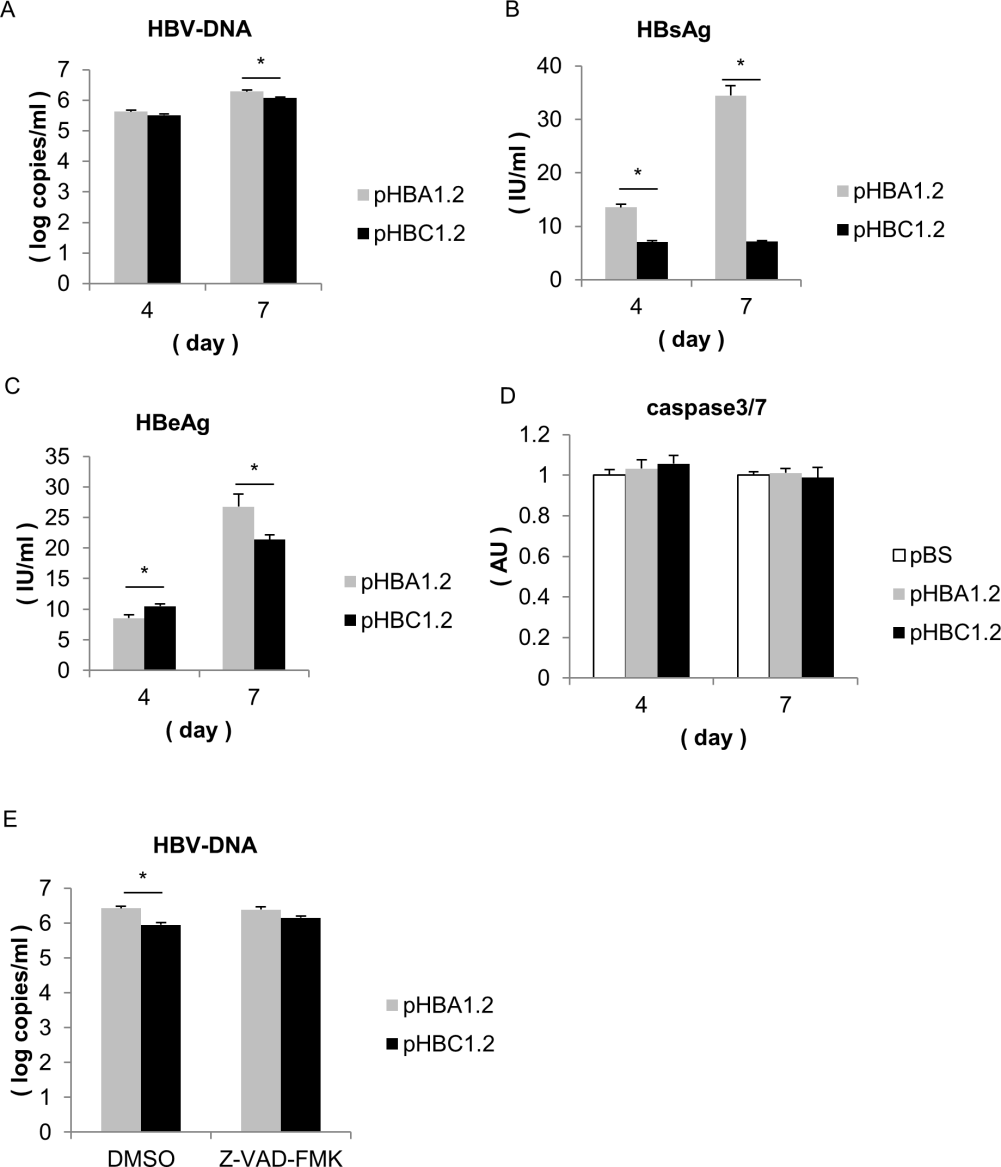
Huh7 cells were transfected with pHBA1.2 or pHBC1.2. **(A-D)** Huh7 cells were transfected with pHBA1.2 or pHBC1.2 in combination with pCMV-SEAP (N = 3). The culture media were collected at days 4 and 7 after transfection. **(A)** HBV DNA levels, **(B)** HBsAg levels **(C)** HbeAg levels and **(D)** Caspase-3/7 activity were assessed in culture medium. **(E)** Huh7 cells were transfected with pHBA1.2 or pHBC1.2 in the absence or presence of Z-VAD-FMK. HBV DNA levels were assessed in culture medium (*, P < 0.05).

To compare HBV genotype A with HBV genotype C, we generated pHBA1.2, with an overlength (1.2-mer) copy of HBV genotype A2 DNA (the adw2 subtype; DDBJ/EMBL/GenBank Accession No.X02763) and pHBC1.2 with an overlength (1.2-mer) copy of HBV genotype C2 DNA (the adr4 subtype; DDBJ/EMBL/GenBank Accession No. LC090200). We confirmed that the transfection efficiency of pHBA1.2 was similar to that of pHBC1.2 by measuring ALP levels in the medium 1 day after co-transfection of the HBV vectors with the SEAP vector into Huh7 cells. HBV DNA was detected in the medium 4 days after co-transfection of the HBV vector with the SEAP vector; however, no difference in HBV DNA levels was detected between pHBA1.2 and pHBC1.2 (Fig 1A). HBsAg and HBeAg were also detected in the culture media of the transfected cells. While the HBsAg levels in pHBA1.2-transfected cells were higher compared to those of pHBC1.2-transfected cells (Fig 1B), the HBeAg levels in the pHBA1.2-transfected cells tended to be lower compared to those of pHBC1.2-transfected cells (Fig 1C). Seven days after co-transfection, HBV DNA, HBsAg and HBeAg levels in the pHBC1.2-transfected cells were lower than those of the pHBA1.2-transfected cells. This difference in HBV DNA was not observed in the presence of a caspase inhibitor (Fig 1E). Not only 3 days after transfection but also 7 days after transfection, we could not detect any significant difference in caspase-3/7 activity between the two groups (Fig 1D).

### HBV genotype A-transfected but not genotype C-transfected immunodeficient mice develop persistent HBV DNA viremia

**Fig 2:**
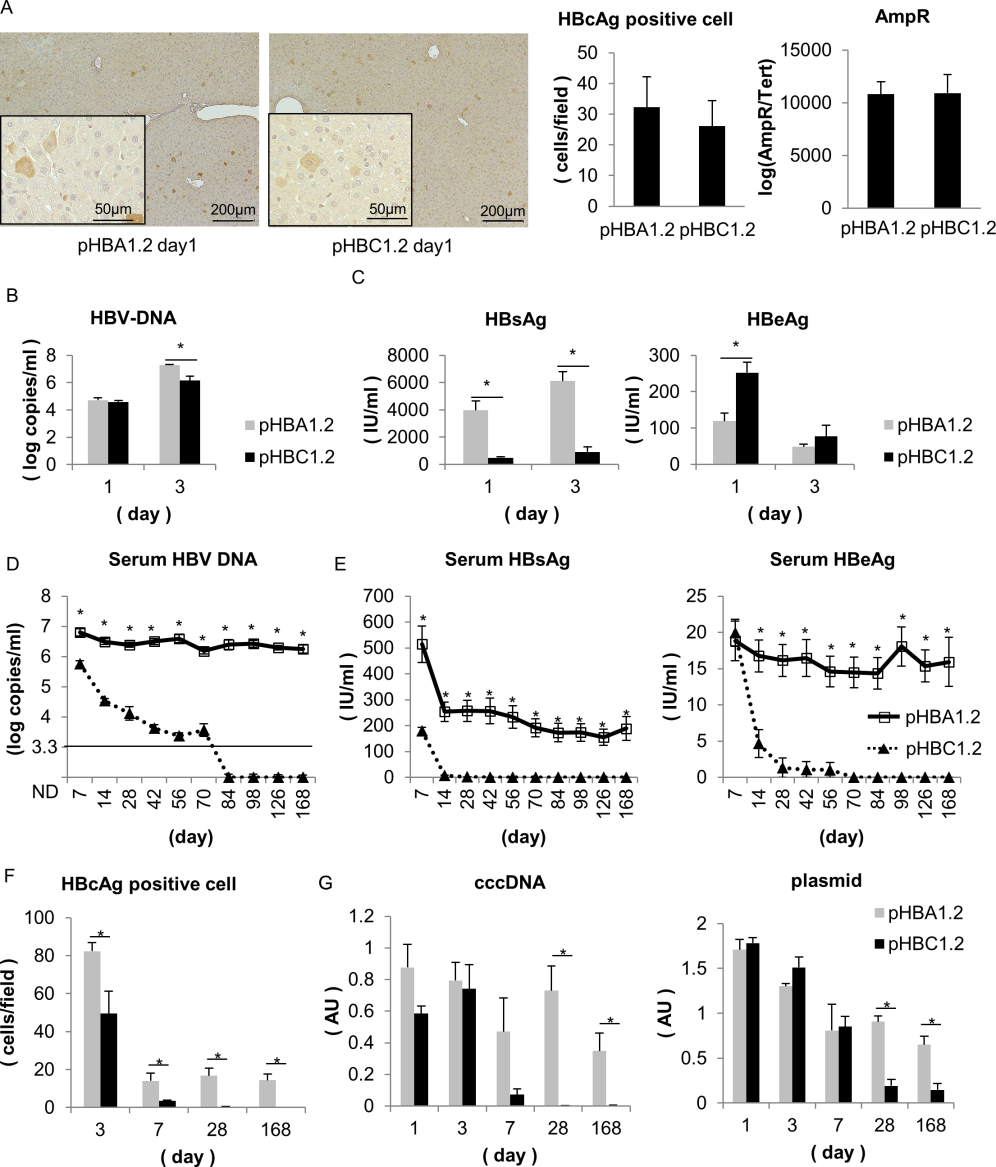
HBV expression in NOG mice transfected with pHBA1.2 or pHBC1.2. **(A-C)** NOG mice were transfected with pHBA1.2 or pHBC1.2 by hydrodynamic injection and analyzed 1 and 3 days after transfection. **(A)** Representative images of immunohistochemical staining for HBcAg in liver sections 1 day after hydrodynamic injection. The number of HBcAg-positive cells in liver sections and the plasmid levels in the livers (N = 9–10). **(B)** Serum HBV DNA levels, **(C)** HBsAg levels and HBeAg levels 1 day after injection (N = 9–10) and 3 days after injection (N = 4). **(D-E)** NOG mice were transfected with pHBA1.2 or pHBC1.2, and blood was collected at the indicated time points. Open squares stands for mice with pHBA1.2, while closed triangles stands for mice with pHBC1.2. **(D)** The serum levels of HBV DNA (N = 7–10). **(E)** The serum levels of HBsAg and HBeAg (N = 7–10). **(F-G)** NOG mice were transfected with pHBA1.2 or pHBC1.2 and analyzed at the indicated time points post-transfection. Gray bars stands for mice with pHBA1.2, and black bars stands for mice with pHBC1.2. **(F)** The number of HBcAg-positive cells in the liver sections was determined by immunohistochemical staining. They were subjected to the Mann–Whitney U-test. (N = 5–7). **(G)** CccDNA and plasmid DNA levels in livers at each time point (N = 4–7). *, P < 0.05.

To evaluate the expression of HBV DNA for long durations after transfection, we transfected pHBA1.2 or pHBC1.2 into mice by hydrodynamic injection. Because we and others have reported previously that immunodeficient mice transfected with an HBV genotype A or genotype D vector developed persistent HBV viremia [23, 24], we used NOG mice on the NOD mice background that are deficient in B cells, T cells and NK cells. One day after hydrodynamic injection, there was no difference in the number of HBcAg-positive cells by immunohistochemistry, in plasmid DNA levels in the liver, or in serum HBV DNA levels between pHBA1.2 and pHBC1.2, suggesting that the transfection efficiencies of the expression vectors *in vivo* were similar (Fig 2A and 2B). Consistent with the *in vitro* results, serum HBsAg levels in pHBA1.2-transfected mice were higher than in pHBC1.2-transfected mice (Fig 2C), whereas the serum HBeAg levels in pHBA1.2-transfected mice were lower than in the pHBC1.2-transfected mice 1 day after hydrodynamic injection (Fig 2C). Serum HBV DNA, HBsAg and HBeAg were detected in the pHBA1.2-transfected mice as late as 5 months after transfection (Fig 2D and 2E). In contrast, serum HBV DNA in pHBC1.2-transfected mice gradually decreased to undetectable levels 3 months after transfection (Fig 2D). Similarly, the serum HBsAg and HBeAg content decreased to undetectable levels within 3 months in pHBC1.2-transfected mice (Fig 2E). Immunohistochemistry against HBcAg revealed that almost all HBcAg-positive hepatocytes decreased to undetectable levels within 1 month in the pHBC1.2-transfected NOG mice, whereas those in the pHBA1.2-transfected NOG mice were maintained at detectable levels for 5 months (Fig 2F). Moreover, both cccDNA and plasmid vector DNA were decreased in the livers of pHBC1.2-transfected mice compared to pHBA1.2-transfected mice (Fig 2G, S2 and S3 Figs). These results suggest that HBV genotype C-transfected hepatocytes were eliminated even in immunodeficient mice.

### Type I interferon is not involved in the difference in HBV genotype clearance in mice lacking immune cells

**Fig 3:**
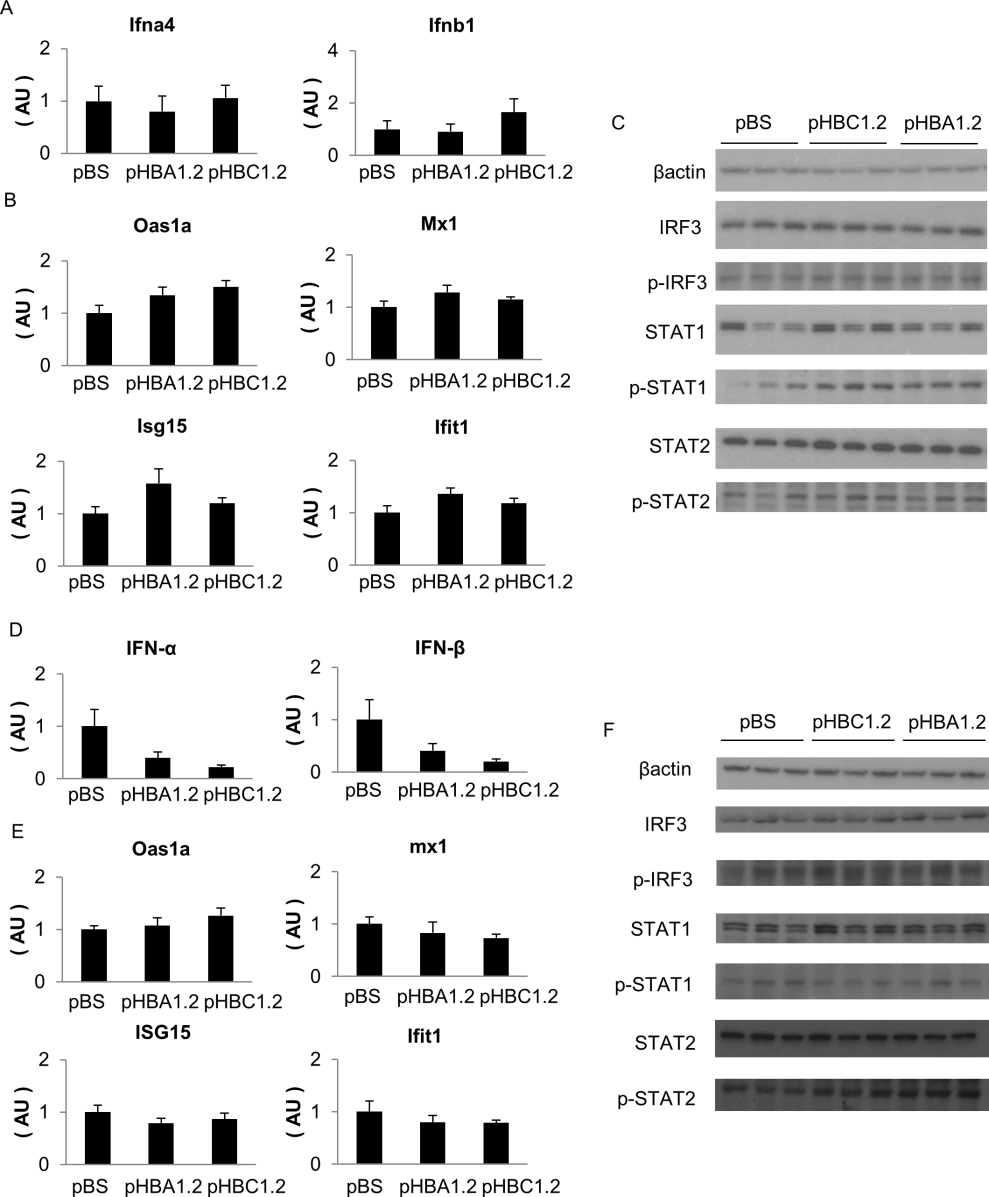
Type 1 interferon signaling in the livers of NOG mice transfected with pHBA1.2 or pHBC1.2. The expression levels of type 1 interferons and ISGs were examined in the livers of NOG mice 3 days **(A-C)** or 7 days **(D-F)** after receiving pHBA1.2 or pHBC1.2 by hydrodynamic injection. **(A, D)** The expression levels of IFN-α4, IFN-β1 and **(B, E)** ISGs (N = 7–8 for 3 days, N = 4 for 7 days). ANOVA was performed to detect any overall difference among the 3 groups. All p values are greater than 0.05. **(C, F)** Western blotting of interferon-signaling related proteins.

HBV interferes with the induction of type 1 interferons that promote viral clearance [14]. However, strong HBV replication was reported to induce type 1 interferon, leading to the suppression of HBV DNA replication [25]. To examine the involvement of type 1 interferon signaling in the clearance of HBV genotype C in the absence of immune cells, we examined the expression levels of IFN-α, IFN-β and interferon-stimulated genes (ISGs) in murine livers. There were no differences in the mRNA levels of type 1 interferons or the ISGs among NOG mice transfected with pBS, pHBA1.2 and pHBC1.2 (Fig 3A, 3B, 3D and 3E). In addition, no difference was observed in the phosphorylation of IRF3, STAT1 or STAT2 (Fig 3C and 3F). Taken together, these results suggest that type 1 interferon signaling was not activated in the HBV-transfected livers of NOG mice. Therefore, the mechanism by which HBV genotype C was cleared in the absence of immune cells was suggested to be independent of type 1 interferon signaling in the liver.

### Hepatocyte apoptosis is increased in the livers of HBV genotype C-transfected NOG mice

**Fig 4:**
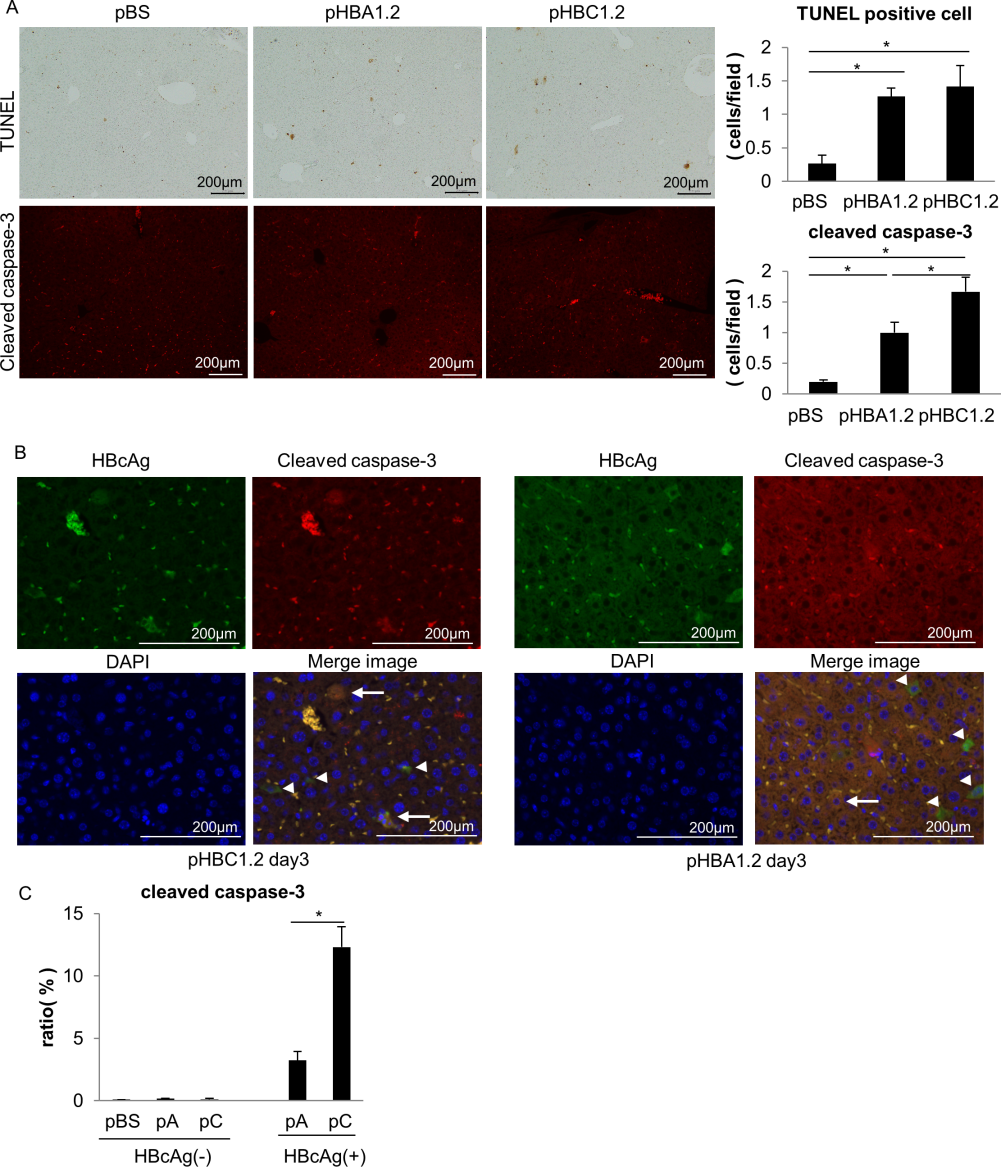
Hepatocyte apoptosis in the livers of NOG mice 3 days post-transfection with pHBA1.2 or pHBC1.2. Liver sections from NOG mice 3 days after transfection with pHBA1.2 or pHBC1.2 were subjected to immunohistochemical staining. **(A)** Representative images of immunohistochemical staining for TUNEL and cleaved caspase-3, and the number of positive cells (N = 4–5). **(B)** Representative images of pHBA1.2-transfected or pHBC1.2-transfected livers with double immunofluorescence staining for the HBc protein (green) and cleaved caspase-3 (red). The arrows indicate HBc and cleaved caspase-3 double-positive cells, while the arrowheads indicate HBc-positive and cleaved caspase-3-negative cells. **(C)** The ratio of cleaved caspase-3-positive cells in HBcAg-negative or -positive hepatocytes (right panel) (N = 4–5). pA: pHBA1.2, pC: pHBC1.2. *, P < 0.05.

Since hepatocyte apoptosis was reported to be induced by an HBV component [26, 27], we hypothesized that HBV genotype A and C expression would induce different levels of hepatocyte apoptosis. To investigate this possibility, we examined hepatocyte apoptosis in the HBV-transfected livers of immunodeficient NOG mice. Although we could not detect any increase in serum ALT levels, TUNEL-positive hepatocytes and cleaved caspase-3-stained hepatocytes were increased in the livers of NOG mice transfected with pHBA1.2 or pHBC1.2 compared with NOG mice transfected with pBS 3 days post-transfection (Fig 4A). This finding suggests that HBV expression indeed caused hepatocyte apoptosis. The number of cleaved caspase-3-stained hepatocytes in pHBC1.2-transfected livers was higher compared with pHBA1.2-transfected livers (Fig 4A). Because more HBV-transfected hepatocytes existed in pHBA1.2-transfected livers compared with pHBC1.2-transfected livers 3 days after transfection (as evidenced by the number of HBcAg-positive cells; Fig 2F), we examined the frequency of apoptotic cells among HBV-transfected hepatocytes. To this end, liver sections were simultaneously stained with HBcAg and cleaved caspase-3 (Fig 4B). Most of the cleaved caspase-3-stained hepatocytes were stained with HBcAg. The frequencies of cleaved caspase-3-positive hepatocytes among HBcAg-negative hepatocytes were quite low and did not differ among livers of mice transfected with pBS, pHBA1.2 and pHBC1.2 (Fig 4C). In contrast, the ratio of cleaved caspase-3-stained hepatocytes among the HBcAg-positive hepatocytes was higher in pHBC1.2-transfected livers than in pHBA1.2-transfected livers (Fig 4C). Collectively, these results suggest that hepatocyte apoptosis was more frequently induced by HBV genotype C compared with genotype A. This finding may contribute to the difference in HBV DNA levels and persistence between HBV genotypes A and C.

## Discussion

In this study, we demonstrated that HBV genotype A-transfected NOG mice developed persistent viremia, whereas the HBV DNA levels in HBV genotype C-transfected NOG mice decreased to undetectable levels within 3 months. This was somehow surprising because many past studies which examined HBV replication in immunodeficient mice transfected by hydrodynamic injection always showed viral persistence [23, 24]. These reports employed an HBV genotype A or genotype D construct. The present study, for the first time, examined HBV replication in immunodeficient mice using plasmid of HBV genotype C and revealed that NOG mice transfected with HBV genotype C by hydrodynamic injection failed to exhibit persistent viremia. Because we used only one strain of HBV genotype C in this study, we cannot exclude the possibility that the observed difference in HBV clearance may be due to the HBV strain used rather than the genotypes. However, this study clearly demonstrates the existence of a mechanism by which HBV-expressing cells were eliminated, even in an immunodeficient context.

Immune cells, such as CD4^+^ T cells, CD8^+^ T cells and NK cells, were reported to be responsible for HBV elimination [13]. Moreover, the immune system was demonstrated to contribute to differences in replication and elimination between HBV genotypes [15, 16]. However, our results clearly show the existence of a mechanism that causes differences in HBV expression independent of the immune system. In this report, although we could not detect any difference of caspase-3/7 activity *in vitro*, we detected differences in the number of cleaved caspase-3-positive hepatocytes in HBV-transfected livers. However, the difference in apoptosis *in vitro* may be too minor to detect by cleaved caspase-3/7 activity. The observation that the difference in the HBV -DNA level between HBV genotype A and genotype C disappeared 7 days after transfection *in vitro* in the presence of a caspase inhibitor supports the idea that apoptosis is involved in the difference in HBV expression level. Ebert et al. [27, 28] reported that the inhibition of apoptosis prevented the clearance of HBV, whereas an antagonizing cellular apoptosis inhibitor promoted the clearance of HBV. Our results indicated that apoptosis would be induced more frequently in hepatocytes expressing HBV genotype C compared to those expressing genotype A, resulting in the elimination of hepatocytes expressing HBV genotype C. Although hepatocyte apoptosis induced an increase in ALT [29, 30], we could not detect an increase in ALT in mice expressing either HBV genotype 3 days after transfection or at later time points. We speculate that the HBV-transfected cells were few and that the number of HBV-expressing hepatocytes undergoing apoptosis was quite small, resulting in an inability to detect changes in serum ALT levels.

In this study, we were unable to clarify the mechanism by which HBV expression led to apoptosis. Many researchers have reported that the HBx protein induces hepatocyte apoptosis [26, 31, 32]. Additionally, the hepatitis B spliced protein (HBSP) induces apoptosis, and its expression levels are different between viral genotypes [33]. The truncated middle hepatitis B surface protein sensitizes cells to apoptosis [34]. Suppression of the preS2 protein downregulates hepatocyte apoptosis [35], whereas the preS1/2 protein downregulates FADD-like interleukin-1β-converting enzyme-inhibitory protein (FLIP) and leads to apoptosis [36]. Conversely, the HBc protein inhibits hepatocyte apoptosis [37]. Thus, various HBV proteins regulate hepatocyte apoptosis. Taken together, differences in the expression levels of these proteins may contribute to the differences in apoptosis. Further study is needed to evaluate these findings.

## Conclusions

Immune cell-independent hepatocyte apoptosis is more frequently induced by the expression of HBV genotype C than genotype A. This leads to the failure of persistent viremia for the HBV genotype C but not for genotype A. This difference may contribute to a lower viral load and reduced persistence in patients with acute hepatitis caused by infection with HBV genotype C compared to HBV genotype A.

## Supporting Information

S1 Fig Expression vectors for HBV genotypes A and C. The HBV-expressing plasmid pHBA1.2 was derived from the genotype A2 HBV strain adw2 (DDBJ/EMBL/Gen-Bank accession number X02763). pHBA1.2 was constructed by inserting the 1.2-fold HBV genome into pBluescriptⅡSK+. The HBV-expressing plasmid pHBC1.2 was derived from the genotype C2 HBV strain adr4 (DDBJ/EMBL/GenBank Accession No. LC090200). The pHBC1.2 was constructed by inserting the 1.2-fold HBV genome into pBluescriptⅡSK+.
(TIF)

S2 Fig Gel images of cccDNA and plasmid DNA in liver sections. (A) A standard curve based on the band intensity levels of 12.5–200 ng of extracted DNA from HBV-infected humanized Tk-NOG mice. **(B)** A representative image of cccDNA (1320 bp) and plasmid DNA (4910 bp) bands on an agarose gel. M: 1 kb ladder marker, DW: distilled water, NG: NOG mouse liver, PtA: patient’s serum with HBV genotype A, PtC: patient’s serum with HBV genotype C, GP: NOG pBluescriptⅡsk+ liver on day 3, GA: NOG pHBA1.2 liver on day 3, GC: NOG pHBC1.2 liver on day 3, TK: liver of humanized-Tk-NOG mouse infected with HBV genotype C, pBS: pBluescriptⅡsk+ plasmid, gA: pHBA1.2 plasmid, gC: pHBC1.2 plasmid. **(C)** Original gel images of cccDNA and plasmid DNA in the livers at each time point shown in Fig 2G. M: 1 kb ladder marker, TK: liver of humanized-Tk-NOG mouse infected with HBV genotype C, NG: NOG mouse liver, DW: distilled water. **(D)** The cccDNA and plasmid DNA levels in the livers at the indicated time points post-transfection with HBV genotypes A or C. M: 1 kb ladder marker.
(TIF)

S3 Fig The persistence of plasmid DNA levels measured by the real-time RT-PCR method. The levels of the ampicillin resistance gene in pBluescriptⅡSK+ were examined in NOG mice at the indicated time points. The results were normalized to the Tert gene (N = 4–7) *, P < 0.05.
(TIF)

## Abbreviations

HBV: Hepatitis B virus
HBsAg: Hepatitis B surface antigen
HBeAg: Hepatitis B e antigen
HBcAg: Hepatitis B core antigen
SEAP: secreted alkaline phosphatase
ALP: alkaline phosphatase
TUNEL: terminal deoxynucleotidyl transferase-mediated deoxyuridine triphosphate nick-end labeling
cccDNA: covalently closed circular DNA
ISGs: interferon-stimulated genes
