# Supplementary figures and images for: The Hepatitis B Virus Genotype Affects the Persistence of Viral Replication in Immunodeficient NOG Mice
Source: PLoS One. 2015 Dec 14;10(12):e0144775. doi: 10.1371/journal.pone.0144775 (PMC4682774; doi:10.1371/journal.pone.0144775)

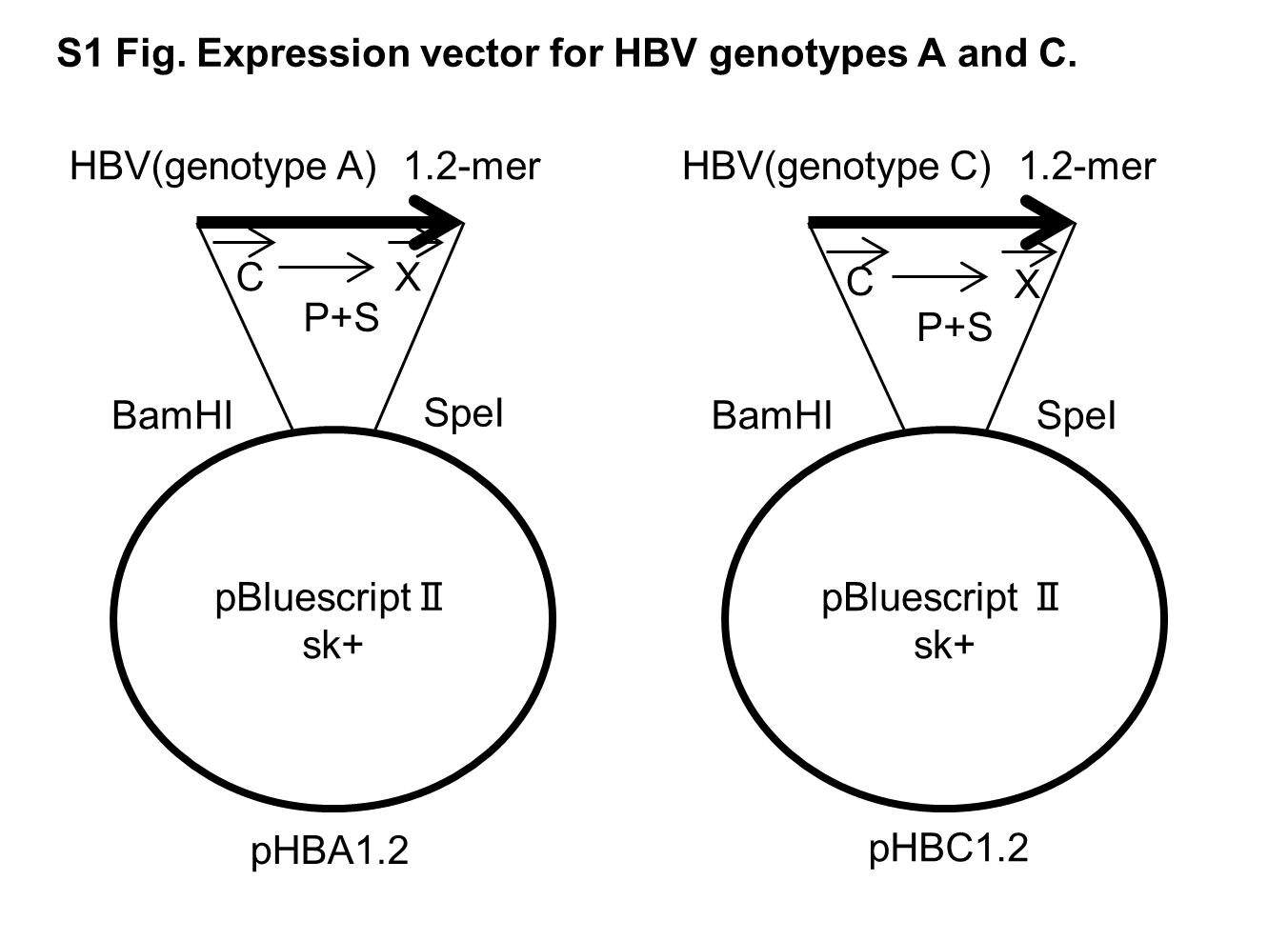

Supplement: S1 Fig — The HBV-expressing plasmid pHBA1.2 was derived from the genotype A2 HBV strain adw2 (DDBJ/EMBL/Gen-Bank accession number X02763). pHBA1.2 was constructed by inserting the 1.2-fold HBV genome into pBluescriptⅡSK+. The HBV-expressing plasmid pHBC1.2 was derived from the genotype C2 HBV strain adr4 (DDBJ/EMBL/GenBank Accession No. LC090200). The pHBC1.2 was constructed by inserting the 1.2-fold HBV genome into pBluescriptⅡSK+. (TIF) [file pone.0144775.s001.tif]

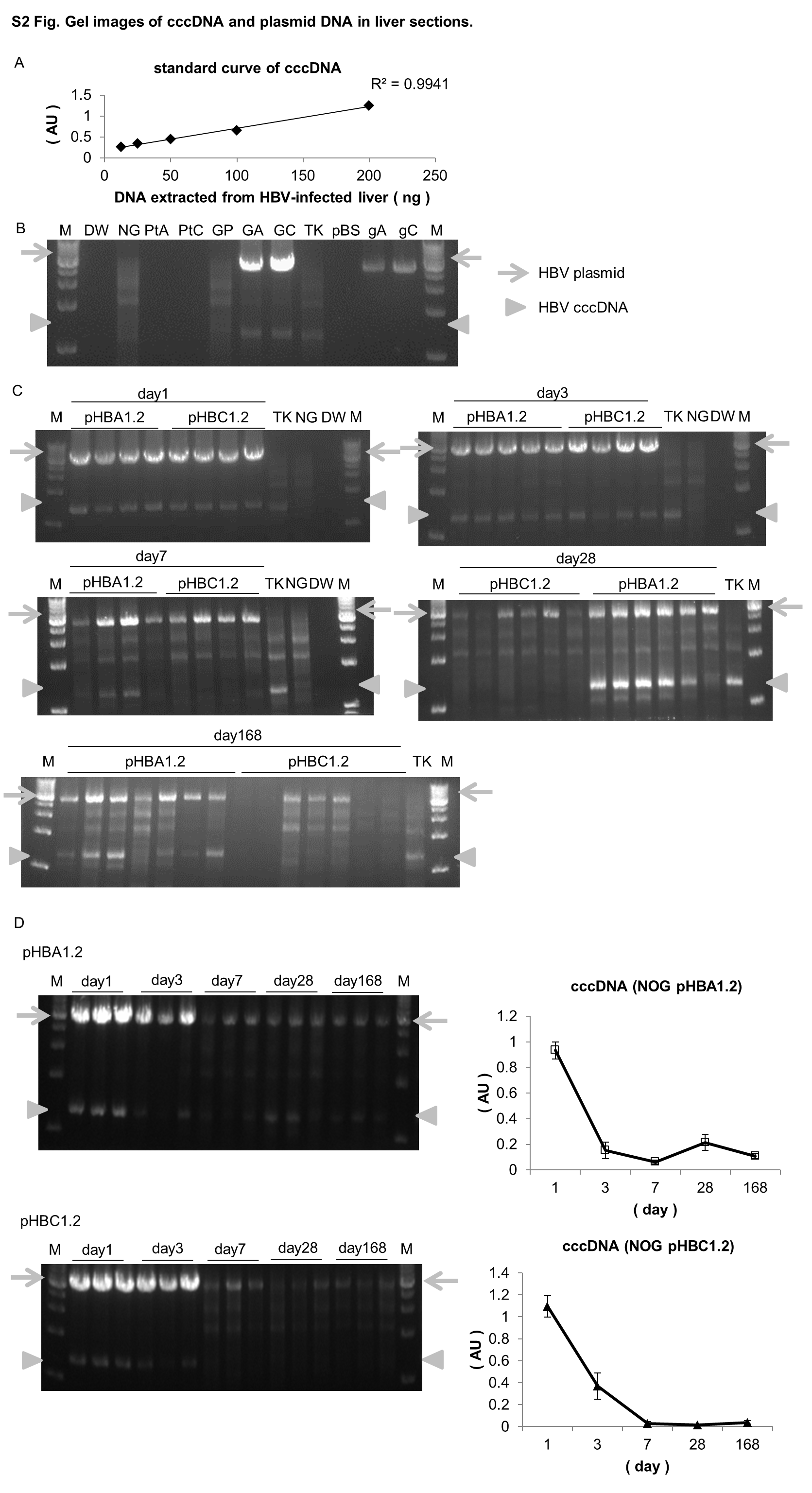

Supplement: S2 Fig — (A) A standard curve based on the band intensity levels of 12.5–200 ng of extracted DNA from HBV-infected humanized Tk-NOG mice. (B) A representative image of cccDNA (1320 bp) and plasmid DNA (4910 bp) bands on an agarose gel. M: 1 kb ladder marker, DW: distilled water, NG: NOG mouse liver, PtA: patient’s serum with HBV genotype A, PtC: patient’s serum with HBV genotype C, GP: NOG pBluescriptⅡsk+ liver on day 3, GA: NOG pHBA1.2 liver on day 3, GC: NOG pHBC1.2 liver on day 3, TK: liver of humanized-Tk-NOG mouse infected with HBV genotype C, pBS: pBluescriptⅡsk+ plasmid, gA: pHBA1.2 plasmid, gC: pHBC1.2 plasmid. (C) Original gel images of cccDNA and plasmid DNA in the livers at each time point shown in Fig 2G. M: 1 kb ladder marker, TK: liver of humanized-Tk-NOG mouse infected with HBV genotype C, NG: NOG mouse liver, DW: distilled water. (D) The cccDNA and plasmid DNA levels in the livers at the indicated time points post-transfection with HBV genotypes A or C. M: 1 kb ladder marker. (TIF) [file pone.0144775.s002.tif]

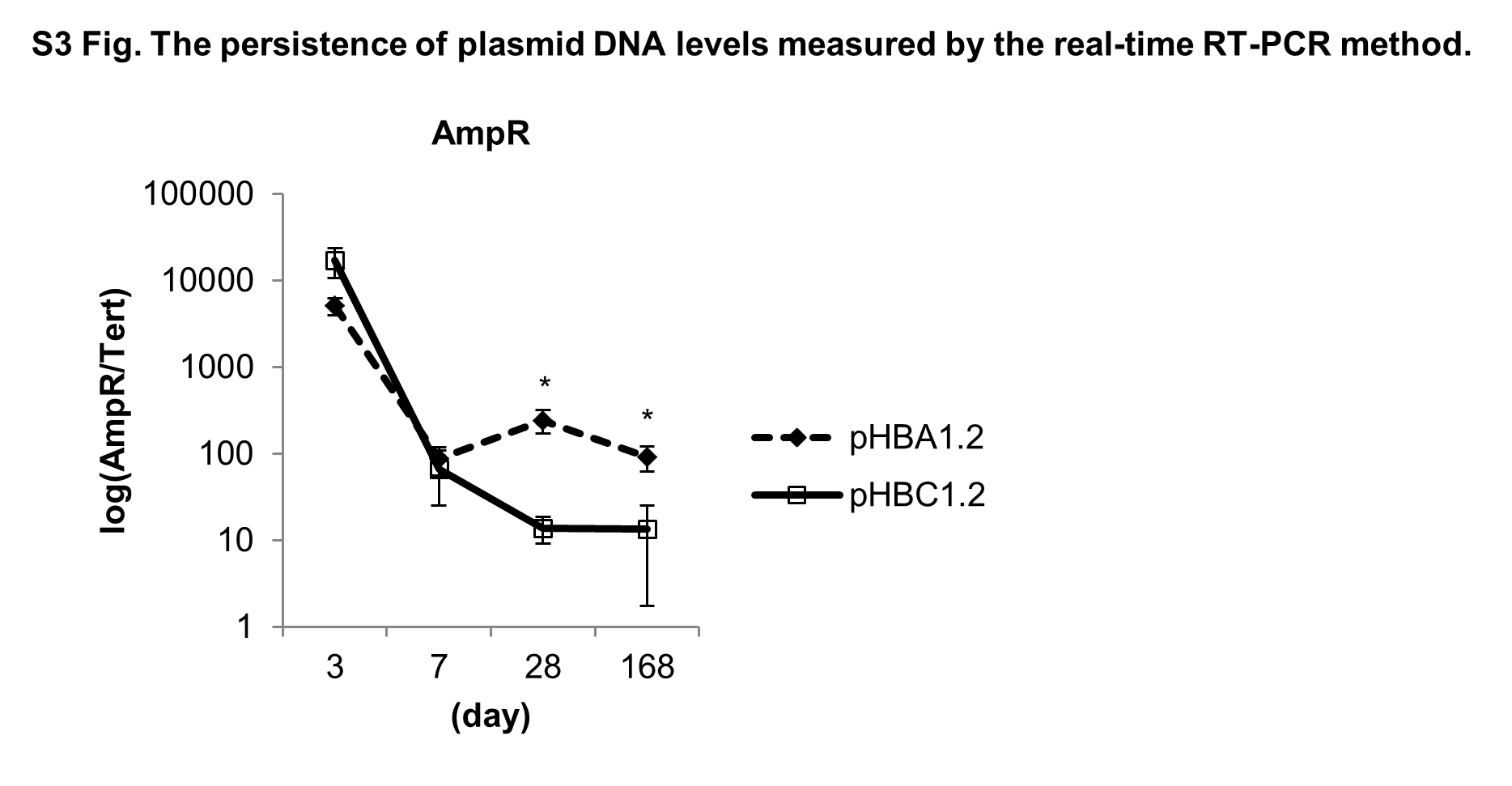

Supplement: S3 Fig — The levels of the ampicillin resistance gene in pBluescriptⅡSK+ were examined in NOG mice at the indicated time points. The results were normalized to the Tert gene (N = 4–7) *, P < 0.05. (TIF) [file pone.0144775.s003.tif]
